# Neurosensory Integration and Balance Adaptation: Personalization of Postural Control Through the Prism of Individual Spatial Perception

**DOI:** 10.3390/life16071125

**Published:** 2026-07-06

**Authors:** Maxim Baltin, Margarita Nikulina, Diana Sabirova, Tatyana Baltina

**Affiliations:** 1 Sirius University of Science and Technology, 354340 Sirius Federal Territory, Russia; margo.nikulina.02@bk.ru (M.N.);; 2 Kazan Federal University, 420008 Kazan, Russia; tvbaltina@gmail.com

**Keywords:** postural control, sensory reweighting, sensorimotor integration, immersive virtual reality, cognitive style

## Abstract

Postural control is a multilevel adaptive system that stabilizes the body in space through dynamic sensory rebalancing and predictive neuromotor regulation. This review synthesizes current data on the neural mechanisms of balance maintenance, individual strategies for integrating multisensory information, and the role of immersive virtual reality as a controlled experimental and neuromodulation platform, synthesising evidence from 73 studies across neuroscience, cognitive psychology, and VR-based rehabilitation. Particular attention is paid to the cognitive style of “field dependence/independence”, which reflects stable preferences for the dominance of visual or vestibular-proprioceptive signals and consistently predicts the selection of postural strategies. We show that traditional averaged models ignore interpersonal variability, whereas immersive VR allows for the parametric induction of sensory conflicts, quantitative assessment of sensory dependence profiles, and facilitates the study of adaptive reorganization at the cortical, brainstem, and spinal levels. The review substantiates the need to move from standardized protocols to personalized approaches in diagnostics and neurorehabilitation that consider individual patterns of input rebalancing. The integration of behavioural metrics with neurophysiological markers in a VR environment provides a foundation for developing predictive balance models and targeted training interventions. However, the translational application of VR-based approaches requires careful consideration of methodological limitations, including cybersickness, hardware latency, and ecological validity, to ensure robust and generalisable outcomes.

## 1. Introduction

Postural control is a fundamental integrative function of the nervous system that maintains vertical body orientation against gravity and enables adaptation to internal and external perturbations. This function relies on the continuous integration of multisensory information and the dynamic tuning of motor responses to task demands. Current models view postural control as arising from the interaction of partially overlapping mechanisms, primarily reactive (feedback-driven) and anticipatory (feedforward) control [1,2].

The coordination of reactive and anticipatory mechanisms depends on the quality and structure of sensory information from the visual, vestibular, and proprioceptive systems. Stability is achieved through sensory reweighting-the dynamic adjustment of the relative contribution of different sensory inputs depending on environmental conditions and task goals [3]. In the presence of sensory conflict or distorted information, the nervous system reweights these inputs, ensuring adaptive postural responses at both rapid reactive and predictive levels.

Current research confirms the importance of sensory reweighting for adapting postural control strategies, demonstrating that motor strategies are substantially modulated by different types of sensory perturbations and form the foundation of dynamic stability adaptation (e.g., distinct muscle activity and kinematic patterns under visual versus somatosensory perturbations) [4]. Moreover, impaired sensory reweighting is linked to reduced postural stability in individuals with neurological conditions, including Parkinson’s disease, where virtual sensory conflict testing reveals significant changes in center-of-pressure displacements and cortical activity compared to controls [5].

Individual differences in sensory reweighting of the postural system reflect variations in the strategy of using visual information, akin to the cognitive styles of field-dependence/independence. In the context of postural control research, field-dependence has been operationalised as a reliance on visual cues for spatial orientation, whereas others rely more on vestibular and proprioceptive signals [6,7]. In the context of postural control, a focus on visual cues or relative independence from them leads to different responses to visual stimuli. For example, field-dependent individuals exhibit more pronounced changes in balance parameters when exposed to a moving visual field, whereas field-independent individuals show less dependence on visual context, relying more consistently on internal sensory signals [8,9]. Such individual differences in visual dependence correlate with postural regulation parameters: the with the nature and magnitude of these changes shaped by individual sensory reweighting strategies. degree to which visual inputs influence center-of-pressure oscillations varies across participants, reflecting diverse sensory strategies in balance tasks and potentially serving as a marker of predisposition to instability under altered sensory conditions.

At the neural level, postural control results from the dynamic integration of sensory information and its transformation into motor commands via a widespread descending control system that includes cortical, subcortical, brainstem, and spinal structures. The cerebral cortex and cerebellum play a critical role in generating anticipatory postural adjustments, providing predictive planning of muscle activations based on internal models of the body and movement [10,11,12]. These structures integrate somatosensory, vestibular, and visual inputs and transform them into anticipatory motor programs that are activated prior to movement initiation, thereby contributing to body stabilization.

Simultaneously, lower-level structures-the brainstem and spinal cord-provide rapid, reactive responses essential for compensating unexpected postural perturbations. The ventromedial descending pathways, primarily the vestibulospinal and reticulospinal tracts, are central to these responses; they receive multisensory inputs and relay commands to motor neurons controlling axial and proximal muscles of the trunk and limbs [1,13].

Furthermore, these ventromedial descending pathways, including the reticulo- and vestibulospinal tracts, perform a key function in modulating spinal reflex excitability. They regulate motor neuron activity through mechanisms such as presynaptic inhibition of Ia-afferents, thereby adapting the contribution of spinal reflexes to postural responses. These pathways receive information from all sensory systems-visual, vestibular, and proprioceptive-and convert it into specific signals for spinal motor neurons. Consequently, the appropriate muscles are activated in the correct sequence and with the required force, ensuring balance and coordinated movement.

While contemporary postural control research actively employs robotic perturbation platforms, computational biomechanical modeling, and non-invasive neuromodulation, immersive virtual reality (VR) offers a unique methodological niche: the capacity to induce parametric, closed-loop sensory conflicts with precise temporal and spatial control. Unlike mechanical perturbation systems that primarily probe reactive motor responses, or static force plates that lack ecological complexity, VR enables independent manipulation of visual flow, spatial reference frames, and multisensory congruence in real time. This allows researchers to isolate and titrate the contribution of specific sensory channels, track dynamic reweighting trajectories, and probe predictive coding mechanisms under ecologically valid yet highly controlled conditions [2]. We therefore selected VR as the focal experimental paradigm for this review because of its unique ability to link behavioural posturography, neurophysiological monitoring, and individualised sensory profiling.

Experimental studies demonstrate that exposure to a virtual environment leads to a reorganization of sensory integration and postural control strategies. VR-modulated balance training with altered visual inputs induces adaptive changes in the use of sensory information and postural response parameters in healthy adults, confirming the influence of VR on sensory reweighting [14,15]. Despite significant progress in applying VR to study and train postural control, substantial gaps remain. First, most studies focus on a single sensory modality or behavioral outcomes without linking sensory reweighting adaptation to correlations at the spinal, brainstem, and cortical levels of control. Second, the role of individual sensory dependence strategies in shaping adaptive responses within a virtual environment is underexplored. Third, understanding of how emotional modulation via VR interacts with the neurophysiological mechanisms of postural control remains limited.

Consequently, we hypothesize that immersive virtual reality induces a multi-level reorganization of postural control, affecting sensory integration and descending motor regulation at the spinal, brainstem, and cortical levels, with the nature and magnitude of these changes modulated by individual sensory reweighting strategies.

Accordingly, this review aims to synthesise current evidence on (1) the neural mechanisms of multi-level sensory reweighting, (2) the role of individual differences such as field-dependence/independence in postural strategy selection, and (3) the application of immersive VR as a controlled platform for personalised neuromodulation.

## 2. Literature Search Strategy

This review is designed as a narrative review aiming to integrate contemporary findings on the neural mechanisms of postural control, individual sensory integration strategies, and the application of immersive virtual reality (VR) as a neuromodulation platform. To ensure methodological transparency, we conducted a structured literature search across three electronic databases: PubMed/MEDLINE, Scopus, and Web of Science Core Collection. We searched publications from January 2000 to 2026, with the start date selected to capture the emergence of modern multisensory integration models and the initial applications of immersive VR in balance research. We restricted the search to English and Russian peer-reviewed articles.

### Search Strategy

A literature search was performed using combinations of the following keywords: “postural control”, “balance”, “standing stability”, “sensory reweighting”, “sensorimotor integration”, “immersive virtual reality”, “cognitive style”, “field dependence”, “field independence”, “spatial perception”, and “sensory conflict”. We combined keywords using standard database syntax (e.g., term1 AND term2) to identify relevant publications across all selected databases. We identified additional studies through manual screening of reference lists of key articles and recent systematic reviews.

Inclusion criteria were studies that: (1) investigated cortical, subcortical, brainstem, or spinal mechanisms underlying postural regulation; (2) examined dynamic sensory reweighting under experimentally induced perturbations or conflicting sensory conditions; (3) used immersive VR or equivalent controlled sensory manipulation paradigms to assess or train balance; (4) addressed individual differences in sensory dependence or cognitive styles (FDI) relevant to postural strategy selection; or (5) were theoretical frameworks, systematic reviews, or meta-analyses directly informing the integration of these domains. We excluded: animal studies; case reports lacking mechanistic or posturographic data; studies focusing exclusively on clinical outcomes without balance or sensory integration metrics; non-peer-reviewed preprints; and publications without accessible full-text methodology.

Screening and eligibility assessment were performed independently by two authors. Given the conceptual and integrative aim of this narrative review, we did not apply formal risk-of-bias assessment or quality scoring Instead, we critically appraised included studies for experimental design rigour, sample characteristics, VR hardware specifications, and relevance to the proposed neurocognitive framework (Table 1).

**Table 1:** Literature search and selection summary.

Stage	Description	Numbers/Criteria
Databases searched	PubMed/MEDLINE, Scopus, Web of Science Core Collection	-
Time frame	January 2000–May 2026	Start date selected to capture emergence of modern multisensory integration models and early VR applications in balance research
Language restrictions	English, Russian	Peer-reviewed articles only
Search string components	(“postural control” OR “balance”) AND (“sensory reweighting” OR “sensorimotor integration”) AND (“virtual reality” OR “sensory conflict”) AND (“field dependence” OR “cognitive style”)	-
Full-text eligibility assessment	Assessment against inclusion/exclusion criteria	Assessed: 118; Excluded: 44 (insufficient methodological detail, no mechanistic data, overlapping publications)
Studies included in synthesis	Narrative, thematic synthesis	73 studies
Thematic distribution of included studies	Categorization by primary focus	• Neural mechanisms: *n* = 24 • Sensory reweighting/FDI: *n* = 21 • VR methodologies: *n* = 19 • Cognitive styles & posture: *n* = 9
Quality appraisal approach	Narrative review design; formal risk-of-bias assessment not applied	Critical appraisal of experimental design, sample characteristics, VR hardware specifications, and relevance to proposed neurocognitive framework

## 3. Modern Theories of Postural Control

### 3.1. Dual Mechanisms of Stability: Reactive and Anticipatory Control

Postural control is currently viewed not as a simple reflex reaction but as a complex sensorimotor system capable of adapting to changing environmental conditions through the dynamic reweighting of sensory inputs and corresponding motor adjustments [3,16]. The main contemporary theoretical frameworks identify two key aspects of stability regulation:

Reactive (feedback)—rapid postural corrections in response to unexpected environmental perturbations.

Anticipatory (feedforward)—predictive postural adjustments that the central nervous system activates before the onset of a predictable movement or perturbation to minimize its destabilizing effect on balance [17].

These two mechanisms operate in close integration, and their relative contribution depends on the speed and predictability of the perturbation. Dynamic sensory reweighting plays a key role in both types of control. In a stable environment, proprioceptive and vestibular inputs predominate; however, when one of them is disrupted (e.g., visual deprivation or soft support), a rapid reassessment of sensory channel weights occurs. Recent research emphasizes that this process is strongly velocity-dependent: during slow tilts of the support surface, proprioceptive information dominates, while during fast tilts, vestibular and visual information prevail [18]. These data indicate that sensory reweighting occurs not only at the cortical level but also at the spinal level through the modulation of presynaptic inhibition of Ia-afferents.

### 3.2. Neural Substrates: A Multi-Level Hierarchical Network

The neural substrates of postural control comprise a distributed, multi-level network that spans cortical, subcortical, brainstem, and spinal structures [1,19,20]. Cortical areas (primary sensorimotor cortex—S1/M1, premotor cortex—PM, and prefrontal cortex) are involved primarily in anticipatory control, multisensory integration, and the formation of predictive motor programs [21]. Subcortical structures, particularly the cerebellum and basal ganglia, enable state estimation, predictive control, and error correction that underlie the adaptation of postural strategies [22]. The brainstem is functionally segmented according to its anatomical and physiological specialisation. The midbrain, containing the red nucleus and superior/inferior colliculi, contributes to rapid orienting responses and audio-visual postural coupling via the rubrospinal and tectospinal tracts. The pontine reticular formation facilitates extensor tone and anticipatory postural adjustments. The medullary reticular formation and vestibular nuclei execute automatic reactive responses. These descending commands primarily travel through the lateral and anterior funiculi of the spinal white matter, coordinating axial and proximal musculature via the vestibulospinal, reticulospinal, and rubrospinal pathways [1,23]. The pyramidal (corticospinal) system is organised somatotopically according to Penfield’s motor and sensory homunculi. It provides voluntary, context-dependent modulation of postural adjustments, particularly during complex or unpredictable tasks. Extrapyramidal pathways from subcortical and brainstem nuclei coordinate automatic balance responses. The red nucleus gives rise to the rubrospinal tract, which contributes to flexor tone modulation and rapid postural corrections. The inferior colliculus (fourth colliculus) mediates audio-motor coupling, enabling rapid orienting and startle-associated postural adjustments. These mechanisms are highly relevant in immersive VR environments, where spatialised auditory cues interact with visual flow. At the spinal level, postural regulation is executed through α- and γ-motoneuron pools. Muscle spindles comprise central non-contractile sensory regions and peripheral contractile poles. The central regions are innervated by group Ia and II afferents to convey length and velocity changes, while the peripheral poles are regulated by γ-motor efferents. This γ-loop mechanism dynamically adjusts spindle sensitivity during stance and movement, allowing descending pathways to modulate proprioceptive gain in accordance with task demands and sensory reliability.

Ascending somatosensory information is organised into distinct functional pathways. These pathways contribute differentially to postural regulation. The dorsal column-medial lemniscus system conveys conscious proprioception, fine tactile discrimination, and vibration sense. These provide high-resolution spatial feedback essential for precise body schema and fine postural adjustments. The spinothalamic (anterolateral) tract transmits nociceptive and thermal signals, which modulate protective withdrawal reflexes and influence attentional allocation during balance challenges. The dorsal and ventral spinocerebellar tracts carry unconscious proprioceptive and load-related feedback directly to the cerebellum. This enables real-time monitoring of limb position, muscle tension, and ongoing movement execution. This functional segregation ensures that the central nervous system can simultaneously process detailed spatial information, detect potential threats, and maintain continuous automatic calibration of posture without cortical overload.

Beyond classical motor pathways, the vestibular cortical network plays a central role in neurosensory integration for balance. Key regions include the parieto-insular vestibular cortex (PIVC), the cingulate sulcus visual area (CSv), and the medial superior temporal area (MSTd). These structures integrate vestibular, visual, and proprioceptive signals to construct an internal model of verticality. They also mediate visual-vestibular interactions, which likely underlie individual differences in spatial perception [24,25,26]. Modern neuroimaging studies have directly mapped these neural substrates. Functional MRI during postural tasks reveals activation in fronto-parietal and vestibular networks. This occurs during anticipatory control and sensory conflict resolution [22,27]. EEG and fNIRS recordings during perturbed stance demonstrate rapid cortical modulation in sensorimotor and prefrontal areas, reflecting dynamic error processing and attentional allocation [11,12,19]. Subcortical structures, particularly the basal ganglia, also contribute critically to postural control. They select appropriate motor programmes, modulate postural tone, and gate sensory information for action selection. Dysfunction in these circuits, as seen in Parkinson’s disease, leads to impaired sensory reweighting, increased reliance on visual cues, and compromised automatic postural responses [5,22,28]. The basal ganglia integrate predictive signals with real-time feedback. This underscores their importance in the adaptive recalibration of balance strategies.

**Figure 1:**
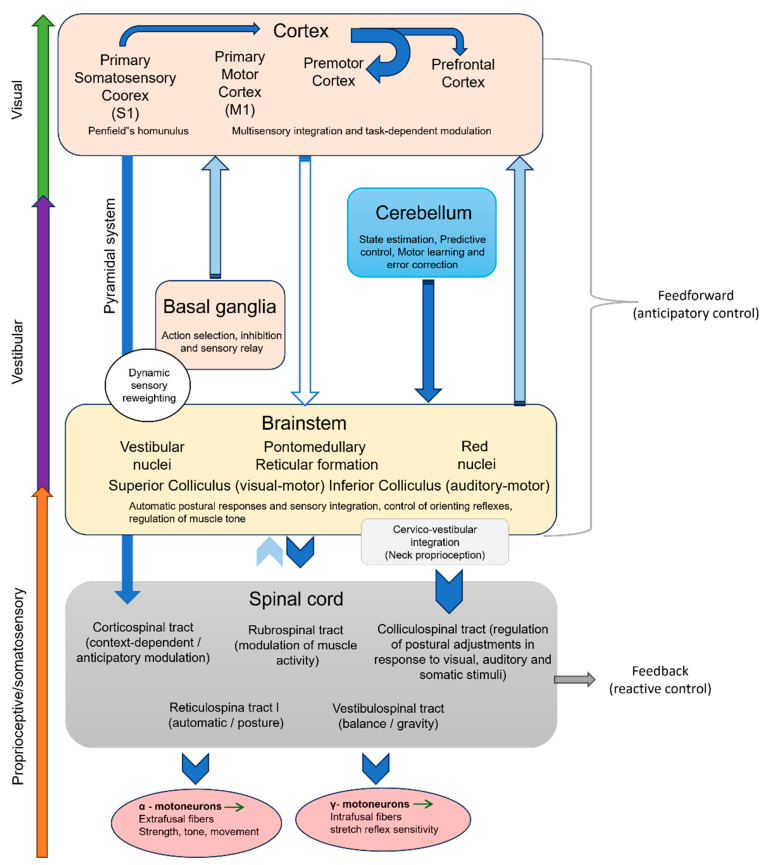
Multi-level neural architecture of postural control. The schematic illustrates the hierarchical integration of cortical (primary somatosensory [S1], primary motor [M1], premotor, and prefrontal cortices, organised somatotopically according to Penfield’s homunculus), cerebellar (vermis, fastigial nucleus), subcortical (basal ganglia, thalamus), and brainstem structures (vestibular nuclei, pontomedullary reticular formation, red nuclei, superior and inferior colliculi). Dark solid arrows denote descending motor pathways (corticospinal, rubrospinal, colliculospinal, reticulospinal, and vestibulospinal tracts). These mediate feedforward (anticipatory) and automatic postural adjustments. Light blue arrows represent ascending sensory feedback and internal state estimation signals. These enable dynamic sensory reweighting and predictive error correction. The spinal level shows the convergence of descending commands on α- and γ-motoneurons. These regulate extrafusal and intrafusal muscle fibres to modulate stretch reflex sensitivity. Colour coding indicates sensory modalities: visual (green), vestibular (purple), and proprioceptive/somatosensory (orange).

As illustrated in Figure 1, postural control emerges from the hierarchical integration of cortical predictive networks, cerebellar error correction, brainstem-mediated automatic reactions, and spinal execution pathways. Bidirectional sensory feedback enables dynamic reweighting according to environmental demands.

Moreover, altered sensory conditions themselves modify postural control strategies in healthy adults. For instance, as task complexity increases, adaptive motor responses (e.g., changes in trunk and limb muscle activity) correlate with the magnitude of change in sensory inputs, reflecting an adaptive reallocation of motor strategies based on which sensory signals are reliable and which are distorted or unavailable [4,29].

A key insight from recent research is the evolved understanding of sensory reweighting not merely as a change in the magnitude of each sensory input’s contribution, but as a cognitively and neurally mediated adaptation of sensory integration in response to perturbation [30]. This goes beyond classical notions of fixed sensorimotor regulation and suggests that the central nervous system continuously evaluates the reliability of sensory inputs and dynamically reweights their contribution to maintain stability. This process aligns with Bayesian integration principles, where the relative weight of each sensory channel is determined by its current accuracy and congruence with other sources of information [31]. The contribution of individual sensory systems is not static and can change under conditions of sensory deficit. Experimental evidence indicates that individuals with hearing impairment exhibit altered stability parameters and increased sensitivity to support surface changes, pointing to an enhanced role of proprioceptive signals in postural control [32,33].

From a neurobiological perspective, sensory reweighting reflects the interaction of distributed networks for sensory integration and motor control, encompassing both subcortical-brainstem and associative cortical structures. Parietal and fronto-parietal associative areas play a special role in constructing an internal representation of the body and spatial orientation. Modern studies using functional connectivity analysis demonstrate that changes in postural strategies are accompanied by a reorganization of cortical networks, including fronto-parietal and sensorimotor circuits, reflecting the adaptive recalibration of sensory integration according to task demands [34].

### 3.3. Experimental and Neurophysiological Methods for Probing Postural Control Mechanisms

The neurophysiological mechanisms underlying postural control are investigated through a multimodal methodological framework. This framework links measurable outputs to specific neural processes. Contemporary research uses distinct experimental paradigms to isolate and quantify specific mechanisms of sensorimotor regulation.

For example, electroencephalography (EEG) and functional near-infrared spectroscopy (fNIRS) capture cortical dynamics during anticipatory and reactive balance tasks. These reveal fronto-parietal network engagement and beta-band desynchronisation, which directly index predictive coding and cortical error processing [11,12,19]. Transcranial magnetic stimulation (TMS) and H-reflex protocols assess corticospinal excitability and spinal presynaptic inhibition of Ia-afferents. These provide direct physiological markers of spinal sensory gating and dynamic reweighting [35]. Vestibular evoked myogenic potentials (VEMPs) and subjective visual vertical (SVV) measurements quantify otolith-vestibular integration and the continuous calibration of the internal model of verticality [24].

High-resolution motion capture, synchronised with force platforms and VR tracking, enables the decomposition of postural sway into rambling and trembling components. It also enables the identification of segmental strategy shifts. This links behavioural adaptation to mechanism-specific motor reprogramming.

### 3.4. Biomechanical Strategies and Segmental Organization

Postural control is implemented not only at the level of the central nervous system but also through the distributed biomechanical organization of the body, where individual segments perform specific functional roles. The body is not a rigid structure; stability is achieved through the coordinated engagement of several segmental strategies. During quiet standing, the so-called “ankle strategy” dominates, where regulation is primarily achieved via activation of distal lower limb muscles, providing anti-gravity support. As perturbation amplitude or speed increases, the contribution of the “hip strategy” grows, involving rapid corrections of the center of mass position by proximal muscle groups. Additionally, movements of the head and shoulder girdle contribute significantly to stabilizing the sensory systems-primarily visual and vestibular-that ensure alignment of the body with the external environment [36,37].

The segmental organization of postural control is tightly linked to sensory regulation, as the effectiveness of each strategy is determined by the availability and reliability of sensory information. Postural maintenance is also governed by the integration of static and statokinetic reflexes. Static reflexes, such as the tonic labyrinthine and neck reflexes, continuously regulate axial muscle tone and head-trunk alignment under steady-state conditions [38]. In contrast, statokinetic (phasic) reflexes generate rapid, compensatory motor responses to dynamic perturbations. The cerebellar vermis plays a pivotal role in coordinating these reflexive networks. It integrates proprioceptive, vestibular, and visual afferentation to fine-tune descending brainstem commands and maintain the internal postural set. Accordingly, the analysis of postural control requires consideration of both motor and sensory mechanisms, which can be purposefully manipulated experimentally. A classic approach involves sensory deprivation or conflict, such as eliminating visual information (eyes closed), distorting proprioceptive signals (unstable or moving support), or altering vestibular inputs, allowing researchers to identify the mechanisms of sensory reweighting and postural strategy adaptation [3,39]. Thus, modern concepts of postural control extend beyond classical reflex models and treat it as a multi-level adaptive system based on the dynamic reweighting of sensory contributions according to their reliability and task context. This process is realized through the interaction of distributed neural networks, including cortical, subcortical, and brainstem structures, and is expressed in the coordinated activity of body segments that form various postural strategies. A key characteristic of this system is its ability to rapidly compensate for sensory disturbances and mechanical constraints by altering both sensory weights and motor patterns. Accumulated experimental evidence demonstrates that postural control is sensitive to targeted manipulations of sensory information and support conditions, which makes it a controllable system [4,14]. Manipulations of sensory inputs and biomechanical constraints lead to predictable changes in stability parameters, reflecting the reweighting of sensory contributions and the reorganization of motor strategies.

## 4. Cognitive Styles of Sensory Integration

### 4.1. Field-Dependence/Independence as a Determinant of Sensory Strategy

Perception of the surrounding space and of one’s own body position within it are not universal processes. The same sensory information can be processed in fundamentally different ways by different individuals, depending on their predominant sensory strategy [40]. This individual difference in sensory integration strategy predicts how the central nervous system constructs postural control in each specific case [8,41]. Depending on the dominant sensory orientation-external (visual) or internal (vestibular-proprioceptive)-different internal models of the body and space are formed, leading to fundamentally different postural strategies even under identical external conditions [9,30].

The classic and still most influential concept explaining individual differences in sensory integration strategies is the theory of cognitive styles field-dependence (FD)/field-independence (FI), developed by Herman Witkin and colleagues in the mid-20th century [40,42]. According to this theory, field-dependent individuals rely heavily on external visual cues when assessing verticality and their own position in space. They are prone to visual capture and demonstrate a pronounced dependence on visual context. Field-independent individuals, conversely, predominantly use internal signals such as vestibular and proprioceptive information, showing relative independence from the visual environment and switching more effectively to other sensory channels when conflicts arise [43,44].

The field-dependence/independence (FDI) construct was originally developed within cognitive psychology to describe perceptual-cognitive styles in visual analysis. It was not intended as a neurophysiological determinant of sensorimotor control.

Modern psychometric research has also noted modest convergent validity across different FDI measures. The Embedded Figures Test (EFT) primarily assesses analytical versus holistic visual processing in a static 2D context. The Rod-and-Frame Test (RFT) evaluates spatial orientation under gravitational conflict. The Body Adjustment Test (BAT) probes reliance on vestibular-proprioceptive cues during whole-body tilts.

These instruments capture related but distinct dimensions of spatial perception. This explains why they do not always yield perfectly overlapping classifications. Nevertheless, within contemporary postural control research, FDI-most frequently operationalised via the EFT-has been consistently adopted as a behavioural proxy for stable sensory integration preferences. It is not used as a rigid cognitive diagnosis.

Empirical studies demonstrate that individuals classified as field-dependent exhibit greater postural sway under moving visual fields, higher visual reliance in sensory conflict paradigms, and altered adaptation curves during VR exposure. Field-independent individuals, by contrast, show greater resilience to visual perturbations and faster recalibration toward internal cues [6,7,8,9,35,43,44,45]. In this context, FDI serves not as a causal neurophysiological driver but as a validated stratification marker. It predicts individual trajectories of sensory reweighting and postural strategy selection.

### 4.2. Diagnostic Approaches: From Embedded Figures to Postural Metrics

The most reliable and widely used diagnostic tool for FDI remains the Gottschaldt Embedded Figures Test (EFT). In this test, the participant must locate a simple geometric figure “hidden” inside a complex one as quickly as possible. Faster and more accurate performance indicates a higher level of field-independence. The field-dependence index is calculated as the ratio of correct answers to completion time. The test possesses high test-retest reliability (r > 0.85) and is actively employed in contemporary studies of postural control, sensory reweighting, and virtual reality [35,46].

The link between FDI cognitive style and postural control is most evident under conditions of sensory deprivation and conflicting sensory paradigms, particularly during the Romberg test and its modifications. The classic integral measure under these conditions is the Romberg quotient, which reflects the relative change in postural stability when visual control is removed.

It has been shown that this quotient is systematically related to individual differences in sensory dependence. Field-dependent individuals are characterized by a marked deterioration in postural stability when transitioning from “eyes open” to “eyes closed” conditions, as well as when proprioceptive information quality is reduced (e.g., standing on a soft surface). This manifests as increased center-of-pressure parameters (velocity, amplitude, and sway area) and reflects the dominance of visual information in forming the spatial-postural model of the body. These patterns align with studies on sensory dependence in postural control and the role of visual “capture” of the postural system [30,47]. In contrast, field-independent individuals demonstrate less sensitivity to visual deprivation and more stable postural control indices during standard Romberg testing. However, under combined sensory conflicts (simultaneous disruption of visual and proprioceptive information, including virtual environments and unstable supports), they may show a more pronounced reorganization of postural strategies, interpreted as a reweighting of sensory contributions driven by a high reliance on internal (vestibular-proprioceptive) signals. These effects are consistent with current views of sensory integration as a context-dependent process sensitive to the quality and consistency of input signals [48].

A comparison of FDI cognitive style with results from the Gottschaldt Embedded Figures Test (EFT) and other spatial perception assessments confirms a robust association between field-independence and more effective postural stabilization. Individuals with high EFT scores exhibit a lower Romberg quotient and less dependence on visual information for maintaining balance, whereas field-dependent individuals display the opposite pattern of sensory organization. Similar findings have been obtained using the Rod-and-Frame Test and computerized dynamic posturography, where cognitive style consistently correlates with sensory reweighting parameters and postural strategy selection [14,46,49].

In the context of modern neurorehabilitation and the use of immersive virtual reality, FDI cognitive style gains particular importance as a predictor of an individual’s response to sensory conflict. Understanding these differences enables a shift from generic postural control models to personalized approaches that account for a person’s unique sensory dependence characteristics.

## 5. Immersive Virtual Reality as a Neuromodulation Tool

### 5.1. Virtual Reality as a Platform for Controlled Sensory Conflict

In recent years, immersive virtual reality has been regarded as an experimental platform that enables controlled modulation of sensorimotor integration and postural control. The use of VR headsets provides a high degree of immersion and precise control over visual afferentation, allowing for the reproducible variation of sensory conditions-something unattainable with traditional stabilometric approaches [2,50]. Unlike classical methods, including sensory organization protocols limited to mechanical and visual distortions in the real world, virtual reality allows for independent manipulation of visual flow parameters, spatial scene configuration, and environmental dynamics, thus creating manageable conflicts between visual, vestibular, and proprioceptive information [51,52].

From the perspective of modern CNS models, postural control is considered a process of minimizing sensory mismatch by continuously calibrating the contribution of various sensory channels. In more recent neurobiological frameworks, this is interpreted as predictive regulation, whereby the CNS forms internal models of the body and environment and compares expected sensory signals with actual incoming ones [53,54]. In immersive VR, a systematic mismatch between predicted and actual sensory inputs arises, increasing sensory error and triggering adaptive sensory reweighting processes. Thus, the impact of the virtual environment is realized not only through altered sensory information but also through modification of the predictive processing mechanisms underlying sensorimotor integration.

Virtual reality artificially increases sensory uncertainty, leading to a reorganization of sensory integration weights and a change in balance maintenance strategies [54,55]. It has been experimentally demonstrated that even brief exposure to an immersive virtual environment can alter the dependence of postural control on visual information and reduce visual dominance.

A key property of virtual reality is the capacity for dosed induction of high-intensity multisensory conflict. Manipulating virtual space parameters (e.g., shifting the horizon, altering the gravitational axis, or introducing scene instability) produces significant changes in postural sway and body stabilization strategy. These effects are accompanied by sensorimotor adaptation, including changes in reaction time and a redistribution of the visual system’s contribution to postural control [51,56].

### 5.2. Neurophysiological Mechanisms of Adaptation in Virtual Environments

At the neurophysiological level, VR’s impact is realized through a multi-level reorganization of sensorimotor regulation. First, a dynamic redistribution of sensory channel contributions is observed, reflecting CNS adaptation to conditions of sensory uncertainty and signal conflict. Experimental data indicate that exposure to an immersive virtual environment alters the dependence of postural control on visual information and reduces its dominant role with repeated exposures [51,57]. Second, VR exposure engages both cortical and subcortical structures that regulate balance. It has been shown that performing tasks in an immersive virtual environment demands greater postural regulation compared to a real environment and is associated with altered sensorimotor processing, reflecting the activation of integrative neural networks that include cerebellar and brainstem postural control mechanisms [58]. Additional evidence suggests that the use of VR in training and rehabilitation protocols is accompanied by changes in the central mechanisms of balance regulation, including structures involved in sensory integration and motor control, indicating the involvement of the cerebellum and brainstem systems in adaptation to the virtual environment [59]. Moreover, several studies have shown that repeated exposure to an immersive virtual environment can lead to lasting changes in postural control strategies. With multiple exposures, a decrease in reliance on visual information and an increase in postural stability under conditions of visual feedback deficit are observed, reflecting the adaptation of sensory reweighting mechanisms and the emergence of more autonomous postural regulation strategies [14,50,60]. These effects have been described in works on VR balance training and sensorimotor adaptation, including studies on reducing visual dominance and improving postural stability after repeated VR sessions.

### 5.3. Age-Related Changes in Sensory Reweighting

Aging profoundly alters the architecture of sensory reweighting. This necessitates dedicated consideration of age-related neuroplastic changes. Older adults typically exhibit reduced vestibular and proprioceptive acuity. This leads to a compensatory shift toward increased visual dependence for postural stability [29,61]. This sensory recalibration is accompanied by structural and functional changes in cortical and subcortical networks. These include reduced grey matter volume in vestibular areas and altered cerebellar connectivity. Consequently, the capacity for dynamic sensory reweighting declines. This results in slower adaptation to environmental perturbations and a higher risk of falls under sensory conflict. These age-related shifts explain why older populations often show exaggerated postural sway in VR environments. They also explain why rehabilitation protocols must account for diminished multisensory integration capacity. This should be done by gradually titrating visual-vestibular conflict intensity.

### 5.4. Individual Variability and the Role of Cognitive Styles in VR Response

It is fundamentally important that the effects of virtual reality on postural control are not universal. The same sensory inputs can evoke significantly different behavioral and neurophysiological responses across individuals, highlighting the pronounced inter-individual variability in spatial regulation mechanisms [57,62,63]. The basis of this variability is the individual manner in which the CNS constructs an internal model of the body’s position in space. Normally, this model is built upon the dynamic integration of visual, vestibular, and proprioceptive information, but the relative importance of these channels differs markedly among individuals [48,64]. In some, the visual system dominates; in others, the vestibular-proprioceptive system prevails, defining the individual sensory perception profile.

This “individual structure of sensory support” predetermines the strategy of spatial body regulation. As a result, some individuals exhibit high resistance to visual distortions and maintain postural stability even under pronounced disruption of visual flow, whereas others experience rapid and significant destabilization from minimal visual changes. In this context, virtual reality acts as an ideal tool that artificially and controllably alters visual conditions, thereby revealing and quantifying individual features of sensory dependence and cognitive styles.

### 5.5. From Universal Protocols to Personalized Neuromodulation

**Figure 2:**
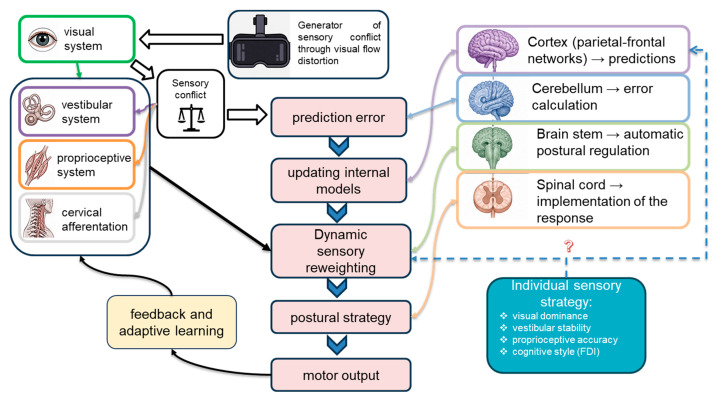
Neurophysiological mechanisms of postural control adaptation in an immersive virtual reality environment. VR-induced visual–vestibular mismatch generates a prediction error that is processed by parieto-frontal networks and the cerebellum, triggering dynamic sensory reweighting and the recalibration of descending brainstem and spinal pathways. The schematic highlights the closed-loop interaction between sensory input, predictive modelling, and motor output under controlled virtual perturbation. Dashed blue arrows represent the top-down modulatory influence of individual sensory strategies (including cognitive style and field-dependence/independence) across the neural hierarchy (cortex, cerebellum, brainstem, and spinal cord). The red question mark indicates the currently unresolved mechanistic interface regarding whether individual sensory reweighting preferences are implemented primarily via cortical gain control or through direct modulation of subcortical and spinal reflex circuits..

Immersive VR creates a controlled sensory conflict by distorting the visual flow while preserving vestibular and proprioceptive afferentation. This mismatch between expected and actual sensory signals generates a prediction error, which triggers the updating of internal models and the dynamic reweighting of sensory contributions (Figure 2).

As depicted in Figure 2, immersive VR creates a controlled sensory conflict. This disrupts the congruence between predicted and actual sensory inputs, generating a prediction error. This error drives adaptive recalibration of sensorimotor integration across cortical, cerebellar, and brainstem-spinal levels.

However, the efficacy of this hierarchical adaptation is not universal; it is substantially modulated by individual characteristics of sensory integration. As discussed in Section 2, cognitive styles such as Field-Dependence/Independence (FDI) determine the baseline preference for specific sensory channels. Consequently, the same virtual sensory conflict can evoke qualitatively different postural strategies in different individuals: field-dependent individuals may exhibit heightened instability due to reliance on distorted visual cues, whereas field-independent individuals may demonstrate faster recalibration relying on preserved proprioceptive inputs [65]. This inter-individual variability highlights the limitations of averaged postural control models and underscores the insufficiency of “one-size-fits-all” rehabilitation protocols.

A critical unresolved question concerns the hierarchical level at which individual sensory strategies modulate postural control. Sensory reweighting has classically been viewed as an automatic, subcortical mechanism driven by signal reliability. However, growing evidence suggests a significant role for top-down cortical modulation. In this framework, the cognitive style of field-dependence/independence acts as a “prior” or cortical gain control that biases the integration process. Specifically, field-dependent individuals may exhibit stronger top-down cortical projections. These maintain high visual weighting even in the presence of sensory conflict, effectively overriding automatic subcortical reweighting signals. Conversely, field-independent individuals may show more efficient cortical gating, allowing rapid down-regulation of unreliable visual inputs. Dynamic sensory reweighting is therefore not merely a passive reflex but an active process. It is modulated by cortical networks that implement individual sensory strategies.

In this context, immersive virtual reality should be viewed not merely as a tool for visual stimulation, but as a precise experimental platform for revealing individual mechanisms of spatial-body model formation. By enabling the systematic modification of visual inputs and the induction of graded sensory conflicts, VR allows for the indirect assessment of sensory reweighting strategies under uncertainty. This capability creates a foundation for transitioning from universal diagnostic methods to personalized approaches in neurorehabilitation. Adapting training interventions to an individual’s specific sensorimotor organization identified through their response to VR-induced conflicts can significantly enhance the effectiveness of restoring postural stability and motor functions. Thus, VR bridges the gap between theoretical models of sensory integration and clinical practice, offering a pathway toward truly personalized neuromodulation.

## 6. Discussion

This review demonstrates that the current understanding of postural control remains fragmented. Sensorimotor models successfully describe the dynamic reweighting of sensory inputs and the multi-level neural organization of stability. Cognitive approaches, in turn, convincingly show the role of individual differences in spatial perception and the selection of postural strategies [1,18,48]. However, the integration of these two lines of inquiry remains insufficient, preventing a full explanation of the observed variability in postural responses, especially under conditions of artificially induced sensory conflict.

A key finding of this analysis is the recognition that individual differences in postural control represent not random variability or measurement noise, but stable strategies of sensorimotor integration. The cognitive style of field-dependence/independence acts here as a behavioural marker that correlates with the configuration of sensory reweighting [14,45,61]. From this perspective, FDI can be considered an intermediate level between cognitive and sensorimotor mechanisms. It potentially reflects the specific ways in which the internal model of the body and space is formed and how sensory uncertainty is resolved. However, at the neural level, this phenomenon remains insufficiently understood. Existing data indicate the involvement of associative parietal areas, the premotor cortex, and the cerebellum in the integration of multisensory information and the formation of predictive models [24,27,66], yet direct links between these networks and cognitive style remain largely hypothetical. This underscores the need to move from behavioral correlations to studies that directly relate cognitive features to parameters of neural activity and functional connectivity.

Immersive virtual reality plays a special role in addressing this issue. In the context of postural control, VR should be regarded not merely as a visualization tool but as a controlled experimental environment for modeling sensory perturbation. Unlike traditional sensory deprivation paradigms, the virtual environment allows for the parametric manipulation of visual information characteristics and the reproducible induction of sensory conflicts of varying complexity [2,67]. This creates a unique opportunity to decompose postural control into its constituent processes, from sensory reweighting to the formation of motor responses.

Critically, virtual reality reveals qualitatively different postural strategies that cannot be reduced to linear changes in stability indices. The same sensory conflict can lead to distinct patterns of adaptation. These differences point to the existence of multiple stable modes of sensorimotor organization, the selection of which is determined by a combination of sensory reliability, cognitive factors, and features of neural regulation. A practical consequence of this approach is the reconsideration of the principles of neurorehabilitation [64,68]. If postural control is indeed individually organized, then the application of uniform training protocols may have limited effectiveness. Conversely, adapting rehabilitation interventions to the individual characteristics of sensorimotor organization-including the sensory reweighting profile and cognitive style-could substantially enhance intervention outcomes. Immersive virtual reality, in this context, represents a promising tool that can not only diagnose the features of postural control but also purposefully modify them through controlled sensory stimulation.

### 6.1. Methodological and Technical Limitations of VR in Postural Research

Immersive VR offers unprecedented control over sensory inputs. However, its application in postural research and rehabilitation is accompanied by important methodological constraints. First, cybersickness-a syndrome of nausea, oculomotor discomfort, and disorientation induced by visual-vestibular mismatch-can independently alter postural sway and attentional allocation. This could confound experimental outcomes [46]. Notably, susceptibility to cybersickness may vary with cognitive style, with field-dependent individuals potentially exhibiting heightened vulnerability due to stronger visual capture [46]. Second, consumer-grade VR headsets introduce visual-motor latency and display-specific artefacts. These can perturb anticipatory postural adjustments, particularly in tasks requiring rapid reactive corrections [52]. Third, current VR systems provide high-fidelity visual perturbation but limited haptic and proprioceptive feedback, restricting the types of sensory conflicts that can be induced [67]. Fourth, the critical question of ecological validity-whether improvements in VR-based balance training transfer to real-world postural stability and fall prevention-remains insufficiently addressed. Evidence across studies is mixed [15,67]. Finally, most existing VR-balance studies employ small sample sizes (typically *n* < 30). This limits statistical power and generalisability to broader clinical populations. Researchers must acknowledge these limitations when designing rigorous future studies. It also helps set realistic expectations for the translational potential of VR-based interventions.

### 6.2. Translational Gaps and Future Directions for Personalized VR Rehabilitation

The framework of individualised sensory reweighting remains conceptual. It requires rigorous empirical validation. To date, no randomised controlled trials have directly compared personalised versus standardised VR protocols for postural rehabilitation. Existing studies predominantly employ fixed-parameter interventions. Consequently, the assumption that personalised approaches outperform standardised ones is currently hypothetical. It represents a priority for future research. Practical implementation also poses significant challenges. VR-based rehabilitation requires trained multidisciplinary teams, standardised hardware specifications (e.g., refresh rate ≥ 90 Hz, latency < 20 ms) [48,69] to minimise technical confounds, and structured protocols for monitoring cybersickness. Cybersickness can independently alter postural sway and attentional allocation. Cost-effectiveness analyses are currently lacking, though the decreasing price of consumer-grade HMDs may gradually improve clinical accessibility.

Transitioning from fixed to adaptive VR protocols requires objective, mechanism-linked biomarkers for monitoring sensory reweighting in real time. Critically, these markers must demonstrate sensitivity to individual sensory dependence profiles and predictive validity for functional outcomes (e.g., fall risk, transfer to real-world balance tasks). Prospective validation of such multimodal biomarker panels is a prerequisite for closed-loop, personalised VR neuromodulation.

A critical limitation of the current framework lies in the construct validity of cognitive style measures when applied to sensorimotor domains. Although FDI correlates robustly with postural outcomes across multiple experimental paradigms, it remains a behavioural proxy rather than a direct neurophysiological determinant. The modest convergent validity between the EFT, RFT, and body-adjustment tasks suggests that “field dependence” may represent a multidimensional construct. It may encompass visual analytical skill, gravitational spatial framing, and vestibular reliance, rather than a unitary trait. Future research should prioritise multimodal validation designs that combine behavioural FDI profiling with neuroimaging (fMRI/EEG), vestibular function testing, and dynamic posturography. This will help disentangle which specific neural circuits mediate the observed sensorimotor preferences. Until such mechanistic links are established, FDI should be interpreted as a practical, psychometrically sound stratification tool for personalised balance assessment. It should not be seen as a standalone explanatory variable for postural control architecture.

### 6.3. Causal Interpretations and Alternative Hypotheses

The evidence linking FDI to postural strategy selection is predominantly correlational. While field-dependent individuals consistently demonstrate greater visual reliance under sensory conflict, this association does not imply direct causality. Three alternative interpretations warrant consideration. First, reverse causality cannot be excluded. Long-term reliance on specific postural strategies (e.g., chronic visual dependence secondary to subtle vestibular hypofunction) may gradually shape perceptual-cognitive styles. Second, both FDI profiles and sensory reweighting tendencies may reflect shared neurobiological substrates, such as the integrity of vestibulo-cerebellar pathways or prefrontal modulation of attention and sensorimotor integration. Third, observed correlations may be task-specific. They may reflect adaptive responses to controlled laboratory perturbations rather than stable traits generalisable to everyday balance behaviour.

The interaction between postural balance and cognitive functions represents a complex, multi-level process. This process changes with age and neurological conditions. Dual-task performance (simultaneous balance maintenance and cognitive task solving) serves as a reliable model for studying this interplay. Within this paradigm, physical activity targeting postural control training has been shown to accompany not only stabilometric improvements but also positive changes in cognitive and psychoemotional domains [25,26,28]. Balance training activates the vestibular apparatus, which closely interacts with the cerebellum, hippocampus, and prefrontal and parietal cortices, potentially influencing cognitive functions such as spatial perception, navigation, memory, and attention [70]. These data indicate that postural control and cognitive functions rely on overlapping neural networks.

Bayot et al. [71] describe that during dual-task walking, not only “direct locomotion pathway” structures (primary motor cortex, cerebellum, spinal cord) but also “indirect pathway” regions (prefrontal cortex, premotor areas, basal ganglia) and fronto-parietal attention networks are activated. Borel and Alescio-Lautier [72] note that cortical involvement in postural control increases with balance impairments, which may explain heightened interference with cognitive tasks in individuals with balance disorders. This aligns with Brauer et al. [73], who demonstrated that balance recovery in older adults with impairments requires greater attentional resources than in healthy peers.

Consequently, based on our cross-sectional design, we can assert only the presence of statistical association. Establishing true causal relationships will require longitudinal studies or experiments with directed manipulation.

### 6.4. Translational Implications for Clinical Populations

The proposed framework of individualised sensory reweighting holds substantial translational relevance for clinical populations where balance deficits are prominent. Beyond Parkinson’s disease, patients with bilateral vestibular loss demonstrate severely impaired sensory reweighting. They rely almost exclusively on residual proprioceptive cues and show poor adaptation to visual-vestibular conflicts. Individuals with cerebellar ataxia exhibit disrupted predictive modelling and error correction. This leads to dysmetric postural adjustments and inefficient reweighting. Post-stroke patients often display asymmetric sensory integration, with hemiparetic sides showing delayed recalibration of visual and proprioceptive weights.

Peripheral neuropathy and age-related sensory decline are characterised by degraded proprioceptive feedback. This forces an over-reliance on visual cues and impairs the nervous system’s ability to dynamically down-weight unreliable inputs. Furthermore, individuals with mild traumatic brain injury often exhibit impaired multisensory integration and delayed sensory reweighting, while patients with normal pressure hydrocephalus display gait and postural instability linked to disrupted periventricular white matter pathways; both populations may benefit from stratified, VR-based sensory reweighting interventions. In such cases, standardised balance training often fails because it does not account for the patient’s altered sensory weighting profile. For these populations, a one-size-fits-all rehabilitation approach is suboptimal. Stratifying patients based on their sensory dependence profile and tailoring VR interventions to gradually challenge their dominant sensory channels could optimise neuroplastic adaptation and functional recovery.

Despite significant progress, several fundamental questions remain open. In particular, the neural mechanisms that determine the selection of a postural strategy under sensory conflict, as well as the nature of the interaction between cognitive and sensorimotor levels of regulation, require further clarification. To date, it remains unclear how individual differences in spatial information processing are translated into specific parameters of sensorimotor integration and postural response.

A promising direction is the shift toward studies that combine behavioral indicators of postural control with neurophysiological markers, including cortical activity, functional connectivity parameters, and measures of spinal excitability. Such an approach would facilitate the transition from descriptive models to a mechanistic understanding of sensory integration processes and their individual variability.

In this context, immersive virtual reality offers a unique experimental platform that enables not only the modeling of sensory conflicts but also the quantitative assessment of individual strategies for spatial regulation under controlled conditions. This opens the possibility of developing predictive models of postural control in which parameters of sensory reweighting and cognitive characteristics can serve as biomarkers of adaptive efficacy.

Future progress will require multicentre studies with standardised VR protocols, larger and more diverse samples, and longitudinal designs. Such designs must assess real-world transfer of VR-induced adaptations. Only through such rigorous methodological frameworks can immersive virtual reality fulfil its promise as a tool for personalised neuromodulation of postural control.

### 6.5. Methodological Limitations

As a narrative review, this manuscript does not follow PRISMA guidelines and does not aim for exhaustive quantitative synthesis. The reliance on thematic relevance and author expertise in study selection inherently carries a risk of selection bias. This may lead to overlooking niche or contradictory findings. The absence of formal quality appraisal means that heterogeneity in VR display parameters, postural outcome measures, and FDI assessment tools is acknowledged. However, it is not statistically pooled. Despite these constraints, the explicit search strategy, transparent inclusion/exclusion criteria, and structured critical synthesis aim to provide a reproducible conceptual framework. This framework bridges sensorimotor neuroscience, cognitive psychology, and personalised VR-based neuromodulation.

## 7. Conclusions

This review highlights that postural control relies on multi-level sensory reweighting characterised by marked individual variability. We conclude that the cognitive style of field-dependence/independence serves as a valuable behavioural marker for stratifying sensory dependence profiles, although its causal status remains unproven and requires further mechanistic validation. Finally, immersive VR offers a powerful, controlled platform for studying and personalising postural rehabilitation, however, its translational application requires rigorous validation through standardised protocols and longitudinal clinical trials.

## Data Availability

Data sharing is not applicable.
